# Plant Invasions in China – Challenges and Chances

**DOI:** 10.1371/journal.pone.0064173

**Published:** 2013-05-14

**Authors:** Jan C. Axmacher, Weiguo Sang

**Affiliations:** 1 UCL Department of Geography, University College London, London, United Kingdom; 2 Key Laboratory of Vegetation and Environmental Change, Institute of Botany, Chinese Academy of Sciences, Beijing, China; University of Konstanz, Germany

## Abstract

Invasive species cause serious environmental and economic harm and threaten global biodiversity. We set out to investigate how quickly invasive plant species are currently spreading in China and how their resulting distribution patterns are linked to socio-economic and environmental conditions. A comparison of the invasive plant species density (log species/log area) reported in 2008 with current data shows that invasive species were originally highly concentrated in the wealthy, southeastern coastal provinces of China, but they are currently rapidly spreading inland. Linear regression models based on the species density and turnover of invasive plants as dependent parameters and principal components representing key socio-economic and environmental parameters as predictors indicate strong positive links between invasive plant density and the overall phytodiversity and associated climatic parameters. Principal components representing socio-economic factors and endemic plant density also show significant positive links with invasive plant density. Urgent control and eradication measures are needed in China's coastal provinces to counteract the rapid inland spread of invasive plants. Strict controls of imports through seaports need to be accompanied by similarly strict controls of the developing horticultural trade and underpinned by awareness campaigns for China's increasingly affluent population to limit the arrival of new invaders. Furthermore, China needs to fully utilize its substantial native phytodiversity, rather than relying on exotics, in current large-scale afforestation projects and in the creation of urban green spaces.

## Introduction

Invasive species form a subset of non-native species which have become naturalized by establishing self-sustaining populations in the natural environment. In addition, invasive species have the ability to permanently spread and colonize large geographic areas [1]. They present a major conservation concern, strongly affecting native communities via competition, predation and disease [2]. The spread of invasive plants also has a severe economic impact in many rural areas, with economic losses expected to further increase in the future [3]. Introduction and subsequent spread of invasive species are commonly assumed to be strongly driven by globalization and the associated increased transfer of goods and people [4]–[5]. Propagules of many invasive plants for example are known to rapidly spread along road networks and anthropogenic waterways [6]–[7].

For more than 2000 years, non-native plants have been introduced and subsequently became naturalized in China [8]. Initially, only a small number of exotic, chiefly Eurasian species established self-sustaining populations, but after the opium wars in the 2^nd^ half of the 19^th^ century, Europeans started to introduce larger numbers of non-native species. Particularly species of American origin subsequently started to spread and colonize increasingly large areas [8]–[9]. The introduction of new plant species in China was further accelerated by the recent economic progress and associated opening of China's economy to the global markets [10].

The exact current number and distribution of invasive plant species in China has proven difficult to establish, as – in contrast for example to the USA or Europe – detailed floristic surveys and distribution maps for individual plant species are not easily available in this large country with its extremely high levels of plant species richness and a high proportion of endemics [11]. Nonetheless, a lot of progress has been made in recent decades, with substantial floras becoming available for the entire country as well as for individual provinces. Furthermore, increasingly detailed accounts of the province-based distribution of invasive plants have recently been published [10], [12]–[17].

A thorough review conducted of published manuscripts and reports, herbaria collections, regional and local floras has nonetheless revealed that the total number of invasive species considered in previous manuscripts, as well as their reported distribution patterns, substantially underrepresents current levels of plant invasions in China. Some of these differences might be a result of increasingly more detailed floristic surveys being conducted throughout China. We nonetheless believe that the huge discrepancies between the data we report on here and earlier studies chiefly reflect the fact that existing invasive plant species have strongly increased their distribution range within China, while some new ones have also recently emerged. The last comprehensive study reporting the known distribution of all invasive plants throughout China [15] relies on data collected prior to 2008. This report is taken as baseline in our study to evaluate the increase in invasive plant species density over the last five years, which we believe provides a good representation of the current speed of spread.

In our study, we firstly aim to determine the extent of recent changes in the distribution of invasive plants in China. In line with the extremely fast socio-economic development experienced by the country and the known close links between the spread of invasive plants and anthropogenic infrastructure, we expect that invasive species are spreading rapidly, resulting in a strong increase in their distribution ranges in the last five years.

We secondly aim to establish the resulting current distribution patterns of invasive plants in China and to identify key environmental and socio-economic parameters associated with these patterns. In light of the current growth of China's economy and international trade, our initial expectation is that socio-economic factors are very important in explaining invasive plant species distribution.

In comparison to invasive species assemblages for example in Europe and the USA, tree species are highly underrepresented in China's invasive species lists. Nonetheless, exotic tree species are widely planted in home gardens as well as in large-scale afforestation projects throughout China. To explore the potential future threat these species pose, we also investigated the current distribution of planted and naturalized populations of non-native tree species in China which are known to be invasive in other parts of the world.

The outcome of our study provides crucial insights for the future management and control of invasive plant species in China and beyond, allowing an evaluation of the chances and challenges we are facing in controlling invasive species in mega-diverse countries.

## Materials and Methods

### Data sources

Our selection of invasive plant species in China conforms to the definition by [1]. We therefore consider all non-native plant species as invasive once they have established naturalized populations in China and subsequently spread over large geographic areas. The resulting invasive plant species list was based on a thorough review of published sources and reports of invasive species from each province, e.g. [10], [12]–[27], as well as on herbaria collections and field visits. For a list of the main data sources on invasive plants in each province, please also refer to [Reference List S1]. Our investigations resulted in the identification of a total of 319 invasive plant species in China [Species List S1]. We additionally established the distribution of non-native tree species growing in China which are known to be invasive either in Europe (DAISIE European Invasive Alien Species Gateway - http://www.europe-aliens.org/) or the USA (Federal Noxious Weed List and State List of Regulated Noxious Weeds, United States Department of Agriculture). While we cannot rule out that differences in botanical research activities reflected for example in the number of botanical gardens and publications on invasive species in individual provinces [Figure S1] might lead to a small underreporting of invasive species from some areas of China, we strongly believe that given the wealth of sources we used to compile our database, the overall trend we report on is very robust.

Socio-economic factors included in this analysis comprise total human population and population density in each province, overall and per-capita Gross Domestic Product (GDP) and overall freight traffic. To describe the state of economic activities, 2010 data was used, whereas the strength of the recent economic development was calculated as the change in the respective figures between 2000 and 2009. Additionally, the percentage area used as cropland in each province in 2009 was included in the analysis. All these parameters were extracted from [28].

Environmental factors considered in this study comprised both biotic and abiotic factors. Overall plant species richness and number of China's endemic plants in each province were used. These figures were based on the authoritative flora for China [29]–[30] as well as on records extracted from regional floras and monographs of endemic plant species, specimen records from the Chinese Virtual Herbarium (http://www.cvh.org.cn/), and further published literature. Each species recorded in China was subsequently classed as native, endemic or invasive, and the latter were further characterized according to their life-form as annual (including biennial) herb, perennial herb, shrub or tree. Net primary productivity (NPP) was included in the analysis as a descriptor of the overall potential for plant growth in each province.

Abiotic environmental factors investigated comprised the overall range in elevation for each province and seven climatic variables. Data on mean annual temperature, mean minimum recorded temperature and the inter-seasonal temperature variation as difference between mean January and July temperature was accompanied by data on mean annual precipitation, precipitation in the warmest quarter and actual as well as potential evapotranspiration. The values of these variables were generated for the geographic centres of each province following the approach outlined by [31]–[32]. Elevation data was based on the USGS Hydro-1K dataset, (http://edcdaac.usgs.gov/gtopo30/hydro/), temperature, precipitation, and evapotranspiration were obtained for the period 1971–2000 from the Chinese Ecosystem Research Network dataset (http://www.cern.ac.cn/0index/), while NPP was based on data from the Socioeconomic Data and Applications Center (SEDAC; http://sedac.ciesin.columbia.edu/es/hanpp.html).

### Data analysis

To account for the substantial differences in area between China's provinces and autonomous regions (in this paper generally referred to as ‘provinces’, which includes all of mainland China, Hainan, as well as Taiwan), species density [ =  log (species number)/log (area)] was calculated for invasive, native and endemic plant species. Furthermore, data for the cities Beijing and Tianjin were combined with data for Hebei Province, for Shanghai with that of Zhejiang Province, for Chongqing with that of Sichuan Province and for both Hong Kong and Macau with data of Guangdong Province. To establish recent trends in the species density of invasive plants in China, the species densities were compared with the data presented by [15].

Both socio-economic and environmental datasets were analysed using Principal Components Analysis (PCA) to establish the main underlying trends in the data. The resulting five principal components (PCs) were subsequently used as predictors in four general linear models based on the overall species density of invasive plant species and the invasive species density of individual life form groups in each province as dependent parameter. The multiple linear regressions were calculated using stepwise forward selection so that principal components with an individual significant contribution (P<0.05) towards the model were included. These calculations were made in STATISTICA 6.0 (Statsoft, Tulsa, UK). The PCs were also used as independent parameters in a Redundancy Analysis (RDA) investigating how the underlying gradients influence the invasive plant species distribution patterns and their spatial turnover. This analysis was conducted with ECOM 1.37 (Pisces Conservation, Lymington, UK).

## Results

### Composition and current distribution of invasive species

The 319 invasive plant species considered in this study represent a total of 200 genera in 58 families. Of these, 49 species (15.4%) had not been recorded as invasive in China in previous studies. The most species-rich genera are *Amaranthus* (13 spp.), *Euphorbia* (10 spp.) and *Solanum* (8 spp.), while representatives of Asteraceae (60 spp.), Poaceae (41 spp.), Fabaceae (27 spp.) and Brassicaceae (22 spp.) dominate the family lists. The vast majority of invasive plants are herbs, which comprise of 161 annual, 16 biennial and 106 perennial species. Due to the overall low number of biennials and their ecological similarity with annuals, these two groups are combined in subsequent analysis. A further 22 species are shrubs, with 14 invasive tree species completing the list. Apart from these 14 invasive tree species, a further 92 non-native tree species occurring in China are listed in invasive and alien species databases for the US and Europe.

**Figure 1:**
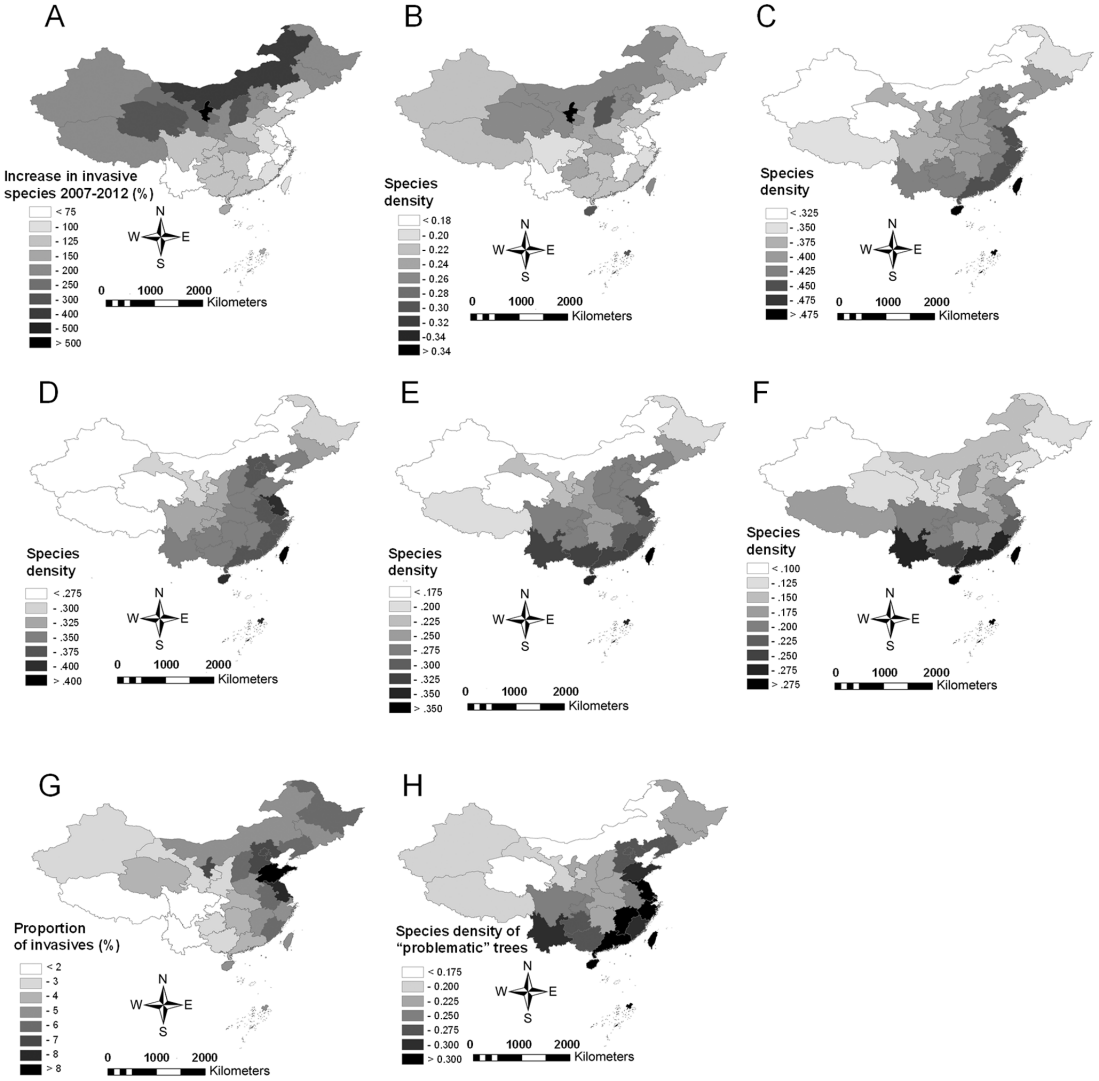
Distribution and recent spread of invasive species. Recent spread and resulting species density (log species richness/log area) and proportion of invasive plant species in China's provinces (A: Increase (%) in the number of invasive plant species in each province between 2007 and 2012, B: Species density of invasive plant species newly recorded in the respective province after 2007, C: species density of all invasive species, D: species density of invasive annual and biennial herbs, E: species density of invasive perennial herbs, F: species density of woody invasive species, G: Contribution (%) of invasive plants towards the total species number in each province, H: Species density of exotic tree species currently not recognized as invasive in China, but reported as invasive species in either the USA or Europe).

Comparisons with the distribution of invasive plant species in 2007 [15] reveal a significant increase in the invasive species density in all provinces, with increases ranging between 65.2% and 800% (Fig. 1A). The strongest proportional increase was observed in the northern and northwestern provinces, where previous records had suggested very low invasion levels. Density patterns of newly recorded invasive plant species (Fig. 1B) show that the strongest increase in invasive plant species density in China occurred in the country's central and Northern provinces, while a significant increase was also observed in Hainan.

The overall distribution of 10% of China's invasive species is currently restricted to a single province, while 30% are reported from less than 5 provinces. On the other hand, 22.6% of the invasive plants have already been recorded from at least 20 provinces [Species List S1]. In the latter group, representatives of the Asteraceae are again dominant, accounting for 13 of these 68 widely distributed species, while Fabaceae and Amaranthaceae follow with 9 species, each. In terms of life-form, 29.8% of all annual invasive plants have a widespread distribution, in comparison to 25% of invasive biennial herbs, 15.1% of invasive perennial herbs, 14.3% of invasive tree and 9.1% of invasive shrub species.

Overall, the highest invasive plant species density occurs at the islands off the Chinese mainland, followed by China's southern and south-eastern coastal provinces, while the lowest density is recorded from provinces in the northwest (Fig. 1C). Differentiation of these geographic patterns for different life forms reveals some subtle spatial deviations from this pattern. Representatives of all life forms reach very high species density levels on islands off the Chinese mainland. On the mainland itself, annual and biennial herbs (Fig. 1D) have furthermore a high species density in the Eastern coastal provinces, which form part of China's agricultural heartland, while perennial herbs (Fig. 1E) are particularly diverse in provinces along both the eastern and southern coast, and woody invasive species density (Fig. 1F) peaks in China's southern provinces. The ratio between invasive species numbers and the total species richness in each province reveals a distinctly different pattern. The highest proportion of invasive plants is recorded from Shandong province, where these species contribute about 8.2% towards all local plant species. Its neighbouring coastal provinces also harbour floras with a high proportion of invasive species. In contrast, the contribution of invasive plants to the highly diverse floras of China's south-western provinces is very small, with figures below 1.5% in Yunnan (Fig. 1G).

The distribution of non-native tree species currently not classed as ‘invasive’ but with known invasion potential (Fig. 1H) finally reflects a very high concentration of these species in all of China's coastal provinces and in Yunnan. Distinctly lower species densities were again recorded for in the country's northern and western provinces.

### China's main socio-economic and environmental gradients

**Figure 2:**
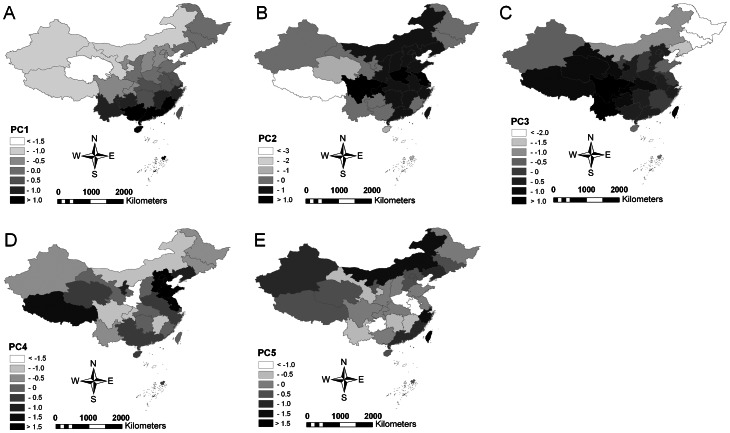
Main socio-economic and environmental gradients in China. Comparison of China's provinces in relation to key socio-economic and environmental gradients, represented as Principal Components (A: PC1 chiefly representing overall plant species density and associated environmental parameters, B: PC2 linked closely to the socio-economic status and cropland proportion of each province, C: PC3 representing species density and proportion of endemic plants and seasonal temperature variation, D: PC4 representing recent changes in socio-economic conditions, E: PC5 mirroring per capita GDP).

The socio-economic and environmental data is associated with five key gradients, represented by five principal components (PCs) in the respective Principal Components Analysis (PCA, see Table 1 for respective factor loadings). The first gradient (38% explained variance) is strongly associated with overall plant species density and most climatic factors. It shows a distinct increase in values from the north-western to south-eastern provinces (Fig. 2A). PC 2 (17% explained variance) chiefly reflects the current economic strength and population density in China and also the proportion of cropland area in each province. High values for this principal component are found in central and eastern parts of the country, with low values recorded both in the western provinces and in China's far north-east (Fig. 2B).

**Table 1:** Factor loadings of environmental and socio-economic parameters on the five principal components (PCs) representing the main socio-economic and environmental gradients encountered in China's provinces.

	Factor loading				
Parameter	PC1	PC2	PC3	PC4	PC5
Species density (all plant species)	***0.78***	0.01	0.52	−0.01	0.18
Mean annual precipitation (mm)	***0.90***	0.19	0.27	−0.04	0.06
Mean summer precipitation (mm)	***0.88***	0.24	0.25	0.05	−0.02
Mean annual temperature (°C)	***0.88***	0.31	0.23	0.11	−0.06
Minimum recorded temperature (°C)	***0.84***	0.21	0.45	0.09	0.03
Potential evapotranspiration (mm)	***0.89***	0.17	−0.18	−0.01	0.05
Actual evapotranspiration (mm)	***0.96***	0.20	0.13	0.02	0.00
Net primary biomass production (NPP)	***0.88***	0.12	0.20	−0.08	−0.06
2010 GDP (RMB)	0.21	***0.90***	−0.04	0.16	0.22
Population density (people/km^2^)	0.56	***0.74***	0.03	0.23	0.01
2010 FREIGHT TRAFFIC (mt)	0.21	***0.93***	−0.03	0.03	0.17
Cropping area (% total area)	0.19	**0.63**	−0.09	0.52	−0.34
Inter-seasonal temperature variation (°C)	−0.60	0.14	−***0.73***	−0.03	−0.21
Species density (endemic species)	0.55	−0.01	***0.82***	−0.11	−0.05
Proportion endemic plants of total flora	0.20	−0.04	***0.89***	−0.16	−0.24
Change in GDP 2000–2009 (%)	−0.03	0.18	−0.18	***0.86***	0.00
Change in population 2000–2009 (%)	0.01	0.24	−0.10	***0.88***	0.05
Change in freight volume 2000–2009 (%)	−0.02	−0.07	0.08	***0.79***	0.36
2010 GDP per capita (RMB)	0.08	0.44	−0.15	0.21	***0.83***
Elevation range (m)	−0.53	−0.44	0.47	−0.43	0.06
Explained variance (%)	38	17	15	14	6
					

Bold numbers in italics indicate a loading>|0.70|, bold numbers indicate a loading>|0.60| on the respective PC, reflecting a good representation of changes in this parameter by the respective principal component.

The third PC (15% explained variance) mirrors changes in both species density and proportion of endemic species in each province and a decrease in inter-seasonal temperature variations. Highest levels of endemic plants are found in Sichuan and Yunnan provinces, while low levels are recorded from northern and particularly north-eastern provinces (Fig. 2C). PC 4 (14% explained variance) reflects change in key socio-economic parameters between 2000 and 2009. High rates of development occurred particularly in the vicinity of the capital Beijing, and in Xizang (Tibet) (Fig. 2D). PC 5 (6% explained variance) finally is chiefly associated with the per capita GDP in 2010. On China's mainland, high values are recorded for the south-eastern coastal provinces, but also for Inner Mongolia, while values are distinctly lower in the inland eastern provinces (Fig. 2E). The altitudinal range in each province is only weakly associated with the five PCs.

### Links between spread and distribution of invasive plant species and key socio-economic and environmental gradients

The increase in invasive plant species density between 2007 and 2012 was not significantly linked to any of the principal components. Subsequently calculated models investigating the contributions of the original set of parameters showed that a model combining the current human population in each province (β = −1.16, p = 0.0005) and provincial freight volume (β = 0.74, p = 0.017) performed best in predicting the increase in invasive plant species density over the 5 year period (F_(2,25)_ = 9.14, adj. R^2^ = 0.38, P = 0.001).

In all linear regression models predicting the current invasive plant species densities across China's provinces (Table 2, models 1–4), PC1 representing overall plant species diversity and the majority of associated climatic factors was by far the best predictor. In models 1–3 predicting species density of all invasives, annuals/biennals and perennial herbs, the remaining four PCs all contributed significantly. In these models, PC2 which reflected the provinces' socio-economic state was of particularly importance in predicting invasive annual/biennial species density. Model 4 predicting woody invasive plant density only included PC 3 representing endemic species diversity and PC5 representing per capita GDP in addition to PC1. A final model (Table 2, model 5) was calculated to investigate links between the current distribution of potentially invasive exotic tree species and the principal components. Again, PC1 was by far the most important predictor in this model, with significant contributions towards the explained variance also by PCs 2,3 and 5.

**Table 2:** Linear models predicting invasive plant species richness of all invasive species (Model 1), annuals/biennials (Model 2), perennial herbs (Model 3), woody invasives (Model 4) and of non-native tree species with known invasion potential (Model 5) based on the five principal components describing environmental and socio-economic parameters as predictors.

	Model 1	Model 2	Model 3	Model 4	Model 5
	(all species)	(annuals/biennials)	(perennial herbs)	(woody plants)	(exotic trees)
	β (P)	β (P)	β (P)	β (P)	β (P)
**PC1**	0.79 (<0.00001)	0.75 (<0.00001)	0.82 (<0.00001)	0.78 (<0.00001)	0.77 (<0.0001)
**PC2**	0.26 (0.002)	0.41 (0.00002)	0.30 (0.0002)	n.s.*	0.25 (0.014)
**PC3**	0.21 (0.01)	0.17 (0.036)	0.29 (0.0002)	0.43 (<0.00001)	0.28 (0.006)
**PC4**	0.24 (0.004)	0.27 (0.002)	0.16 (0.027)	n.s.*	n.s.*
**PC5**	0.29 (0.0008)	0.20 (0.017)	0.16 (0.025)	0.30 (0.0004)	0.25 (0.014)
**Adjusted R^2^**	0.85	0.84	0.88	0.86	0,76
**F**	31.53	30.00	40.05	56.56	22.57
**Deg. freedom**	5,22	5,22	5,22	3,24	4,23
**P (model)**	<0.00001	<0.00001	<0.00001	<0.00001	<0.00001

* n.s.: not significant.

### Factors affecting the turnover in invasive plant species between provinces

**Figure 3:**
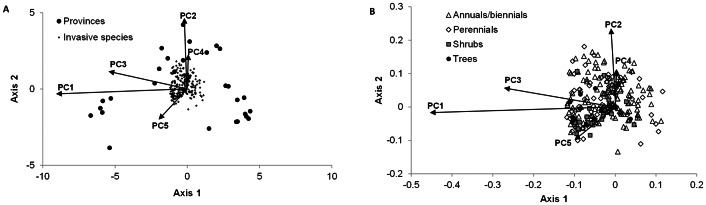
Shifts in invasive species distribution in relation to main socio-economic and environmental gradients. Ordination plot based on the redundancy analysis of invasive plant species distribution and the five principal components representing socio-economic and environmental factors (A: triplot combining principal components (arrows), provinces and invasive species, B: biplot combining principal components and invasive species, differentiated into different life forms).

Similar to the overall number of invasive species, the spatial turnover of invasive species is also primarily influenced by shifts in abiotic conditions and associated changes in overall phytodiversity, which are represented by PC1. In the RDA ordination plot (Fig. 3A), the respective PC shows the longest vector length and is closely aligned with axis 1, which explains 29.1% of the underlying variance. PC3, representing the diversity of endemic plant species, also has a high vector length associated with this axis. Axis 2, representing a further 8.2% of the variance, is closely associated with PC2, reflecting the current socio-economic state and the proportion of cropland area in China's provinces. The remaining two PCs had much shorter vector lengths, showing their smaller contribution towards the shift in invasive species composition between provinces. A separation of species according to their life form (Fig. 3B) shows that herbaceous species are generally distributed along all respective gradients in socio-economic and environmental conditions. Woody plants on the other hand appear to be somewhat more closely associated with areas of high phytodiversity and endemic plant species richness and with a lower current state of economic development.

## Discussion

### Implications of current invasive plant distribution patterns

A first key finding of this study is that despite their observed rapid inland spread, invasive species are currently still highly concentrated in a small number of provinces along China's coastline and at the islands off China's coast. The coastal provinces harbour many of the world's largest seaports, and they have already been identified as key area for the initial introduction of invasive plant species into China [17]. The geographic coincidence between China's main seaports and the hotspot of invasive plant density shows that a strict, targeted control of all goods imported through these ports is needed to counteract the accidental release of any new plant species with invasive potential. To achieve this, a shift from controls targeting species already known to be potentially economically harmful [33] to a thorough assessment of potential future environmental threats posed by any exotic plant material prior to its import is in our view required. This approach could closely follow the example of the weed risk assessment process currently used in Australia.

Secondly and somewhat reassuringly, the concentration of invasive plants along China's coastline suggests that many invasive plant species are still in an early stage of establishment. The current invasive plant distribution pattern is likely to reflect a period of naturalization and adaptation to differences in environmental conditions between native and newly colonized distribution ranges of the invasive plant species [7]–[8], [34]–[35], rather than a long-term limitation to coastal areas. The general lack of environmental barriers limiting the further expansions of invasive plants inland is reflected by the fact that more than 75% of all vascular plant species encountered in the strongly invaded coastal provinces are also found further inland. The initial establishment of small, stable populations of invasive species in the natural environment and their subsequent adaptation to the local conditions of the wider region can take many decades [36]–[38]. It therefore seems highly probable that the inland areas of China will only experience the full impact of current plant invasions in future decades or even centuries – if no effective countermeasures are taken.

Despite the prevailing high concentration of invasive plants along China's south-eastern coastline, our comparative results with invasive plant species distributions in 2007 paint a very alarming picture. Some of the most extreme increases observed might be partly related to more complete surveys and associated provincial floras now being available, and the number of plant species recognized as invasive has also slightly increased. Nonetheless, our records show that the number of invasive plant species has at least doubled in all but four provinces, with the highest increase observed in some of China's northern and western provinces which were previously least affected by invasive plants. This supports our assumption that parallel to the strongly developing economy, the invasive species density is also rapidly increasing throughout the country. This indicates that the window of opportunity during which it might be feasible to limit the extend and speed of the inland spread of invasive plant species via the effective concentration of control and eradication measures in China's south-eastern coastal provinces is closing fast. Once species have continued their spread inland, controlling them will become progressively costly – and their eradication increasingly impossible. Current control and eradication measures must therefore urgently be improved to counteract the increase in invasive plant distribution ranges.

### Risks for the natural environment

The great overall similarity in species density patterns between invasive and native plant species reflected in our models clearly demonstrates that across large geographic regions, both groups are similarly influenced by underlying environmental factors, a trend mirroring previous studies [39]–[44]. This positive relationship leaves China's phytodiversity hotspots highly susceptible to invasions by exotic colonizers. In the long-term, efforts to control invasive plants will therefore need to be increasingly re-focussed on regions of high plant diversity to safeguard China's enormous plant species richness.

The risk invasive plants pose to China's plant species richness is further exacerbated by the significant positive link between invasive woody and perennial plant species densities and the species density of endemic plants. Endemics are typically encountered in areas comprised of isolated, fragmented habitats like high mountain ranges, where they have either formed by allopatric speciation or remain as relic populations shielded from potentially stronger competitors [45]–[46]. A combination of increasing anthropogenic pressure on their habitats and the often small population sizes of endemic plants means that many species are already threatened. Our models suggest that the regions with particularly high levels of endemics are also particularly affected by the additional pressure posed by invasive plants. The safeguarding of the ecosystem integrity in China's remaining isolated patches of natural habitat with high levels of endemic and threatened plant species must therefore become another priority. To prevent the creation of any ‘invasion windows’ [47] through anthropogenic disturbances, for example in relation to the massive increase in tourism experienced by remnant pristine environments [48], anthropogenic activities that potentially cause environmental damage and disturbances should be concentrated in areas of low endemic and overall plant species richness.

Minimizing anthropogenic disturbances is seen as an effective control strategy as particularly many annual invasive herb species, which form by far the biggest group of invasive plant species in China, actually require regular disturbance events to sustain stable populations. Their distribution is therefore currently centred on ruderal and agricultural habitats [13], [15], [19]–[20], [22], [49]. This pattern also explains the strong influence of the PC representing the proportion of cropland area in each province on invasive annuals in our model and their substantial contribution towards the local flora in parts of China's agricultural heartland. Nonetheless, species lists obtained from numerous protected areas clearly show that invasive species have become widespread in a much wider variety of habitats in China, including pristine habitats within protected areas [8], [15].

### Implications of links with socio-economic factors

Contrary to our initial assumptions and suggestions by [10], and somewhat surprising given the predominant role humans play in the initial introduction of exotic species to new geographic areas, our models show only a limited influence of socio-economic factors on the overall current distribution of invasive plants in comparison to abiotic factors related to plant species richness. This might partly be a scale-dependent effect, as well as potentially showing that the initial introduction of many invasive plant species in China occurred in the more distant past, leading to closer links with socio-economic conditions prevailing at their respective time of introduction – a trend also recently reported for Europe [50]. Apart from the aforementioned strong link between invasive species and anthropogenic disturbance regimes, humans also enable invasive species to rapidly expand their distribution range along anthropogenic infrastructure [6]–[7], [34]–[35], [51]–[52]. This is assumed to be at least partly responsible for the significant positive association between invasive species density and both, the current socio-economic status and recent development in China's provinces, but particularly also in explaining the observed recent increase in invasive plant species densities.

An additional, worrying trend underlying the link between economic development and the increasing spread of invasive plants is reflected in the high explanatory value of the gradient describing personal wealth in predicting overall invasive species density. This mirrors a trend in the increasingly affluent population to invest more into horticultural products, with a distinct shift from agricultural produce grown in suburban and rural home gardens to ornamental species [8], [35], [53]. This trend increases the risk that some of the ornamental species become naturalized, with their populations forming a starting point for future invasions. A two-fold approach is required to address this threat. Public campaigns are urgently needed to address the very weak awareness in China's general public about the great economic and environmental risks posed by invasive plants [8], [35]. This needs to be supported by a very strict control of the horticultural trade [35] – both internationally via the aforementioned risk assessment for all exotic plants and plant material prior to its import, as well as domestically, with the respective monitoring within China covering both known invasive species as well as exotic species with invasion potential. Equally important, both the horticultural trade and local administrations responsible for the management and establishment of vegetation need to be strongly encouraged to rely on China's huge internal plant diversity [11]. Ideally, planting of exotic species should mainly be undertaken when no adequate alternative local or regional species is available. This approach should not only be used by administrations responsible for the planting of China's parks and urban green spaces, but also in the establishment and management of pasture land and other grassland habitats [8], [35], and particularly in China's current re- and afforestation programmes, which arguably form the largest ecological restoration project on the planet [54]. At the moment, exotic species are commonly used as monocultures in these plantations [55], posing substantial, long-term risks to the biodiversity of replanted areas. The extensive use of non-native tree species in China's large-scale reforestation projects reflected in the widespread distribution of exotic tree species with known invasion potential in China's provinces might also explain the suspicious overall lack of tree species in the invasive species lists. Currently, the establishment and spread of these exotic trees is likely to be perceived as beneficial for the key aim of the re- and afforestation projects, which is to increase the country's forest cover – while potential long-term threats posed by these species are widely ignored.

### Future challenges

Several trends need to be addressed in relation to the future management of invasive species in China. One pattern clearly emerging is the strong similarity between invasive floras of China, Europe and America. While America is the most important source area of China's invasive plant species [13], [15], [33], [51], [56]–[57], a comparison of China's invasive species with the Federal Noxious Weed List and State List of Regulated Noxious Weeds published by the United States Department of Agriculture still yielded an overlap of more than 20%, while 69% of China's recognized invasive plant species also appear in the European DAISIE (European Invasive Alien Species Gateway - http://www.europe-aliens.org/) database. The substantial body of available literature on invasive plants and their management both in the US and Europe is therefore of direct significance for China's fight against plant invasions, and international cooperation in this sector should be strongly encouraged.

Another important factor which needs to be fully considered in the future management of invasive species is a shift in the potential distribution ranges of invasive plant species associated with climatic change. We have demonstrated that climatic factors associated with overall plant diversity are key drivers of spatial variations in invasive plant species compositions. Changes in climatic conditions are already putting stress on native plant communities, with associated shifts in the community structure allowing new opportunities for invasive species to colonize. This makes the planning of future control measures even more complex. In light of these developments, the restriction of anthropogenic pressures and disturbances in protected areas and phytodiversity hotspots becomes even more important to limit the spread of new invasive species in these areas.

## Supporting Information

Figure S1 **Number of botanical gardens (A) and publications on invasive plant species (B) in China's provinces.**
(TIF)

Reference List S1 **Key references used to determine the distribution of invasive plant species in China's provinces.**
(DOC)

Species List S1 **List of invasive plant species in China.**
(DOC)
