# Supplementary figures and images for: Plant Invasions in China – Challenges and Chances
Source: PLoS One. 2013 May 14;8(5):e64173. doi: 10.1371/journal.pone.0064173 (PMC3653845; doi:10.1371/journal.pone.0064173)

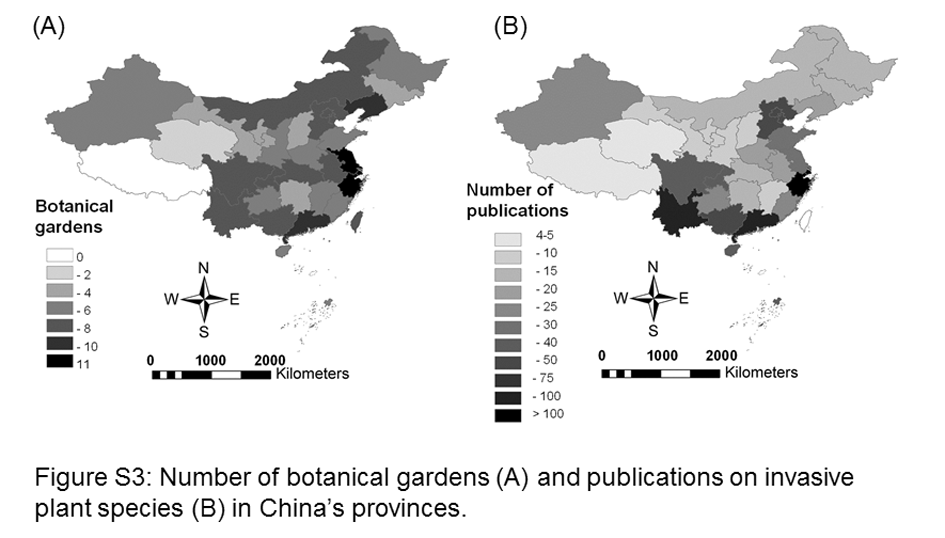

Supplement: Figure S1 — Number of botanical gardens (A) and publications on invasive plant species (B) in China's provinces. (TIF) [file pone.0064173.s001.tif]
