# Supplementary material for: Plant Invasions in China – Challenges and Chances
Source: PLoS One. 2013 May 14;8(5):e64173. doi: 10.1371/journal.pone.0064173 (PMC3653845; doi:10.1371/journal.pone.0064173)
Supplement: Reference List S1 — Key references used to determine the distribution of invasive plant species in China's provinces. (DOC) [file pone.0064173.s002.doc]

**Reference list S1**

**KEY REFERENCES USED TO DETERMINE THE DISTRIBUTION OF INVASIVE PLANT SPECIES IN CHINA’S PROVINCES**

Catalogue of Life: Higher Plants in China (CNPC) ([http://www.cnpc.ac.cn](http://www.cnpc.ac.cn/))

Chinese Virtual Herbarium (CVH) ([http://www.cvh.org.cn](http://www.cvh.org.cn/))

Ding J, Wang R (1998) Invasive alien species and their impact on biodiversity in China. China Environmental Science Press, Beijing.

Feng J, Zhu Y (2010) Alien invasive plants in China: risk assessment and spatial patterns. Biodiversity and Conservation 19: 3489-3497.

Feng M, Dong X, Xu C (2009) Spatial patterns of floristic composition of invasive alien plants in large scale and their climatic interpretation. Journal of Wuhan Botanical Research 27: 159-164.

Guo S, Li Y (1995) Alien weeds in the southeast of China. Weed Science (China) 2: 4-8.

He J, Ge J (2008) The present situation of alien invasive plants in Anhui province and a comparison with the other provinces in China. Journal of Anhui University Natural Science Edition 32: 82-89.

Huang D, Zhang R, Kim KC, Suarez AV (2012) Spatial pattern and determinants of the first detection locations of invasive alien species in mainland China. PLoS ONE 7: e31734, 1-7.

Huang Q, Wang G, Hou Y, Peng S (2011) Distribution of invasive plants in China in relation to geographical origin and life cycle. Weed Research 51: 534-542.

IPP CAAS Database of invasive alien species in China (http://www.agripests.cn/index.asp).

ISSG Global invasive species database (http://www.issg.org/database/welcome/).

IUCN 100 of the World’s Worst Invasive Alien Species (<http://www.issg.org/database/> species/search.asp?st=100ss&fr=1&str=&lang=EN).

Ji C, Wang Z, Qian P (2009) Primary Research on Invasive Plants in Jiangxi Province. Hubei Forestry Science and Technology 127: 26-30.

Jiang H, Fan Q, Li J, Shi S, Li S, Liao WB, Shu W (2011) Naturalization of alien plants in China. Biodiversity and Conservation 20: 1545-1556.

Li B, Hsu P, Chen J (2001) Perspectives on general trends of plant invasions with special reference to alien weed flora of Shanghai. Biodiversity Science 9: 446-457.

Li Y, Yao G, Deng F, Huang G (2008) Preliminary investigation and analysis on exotic spermatophytes in Jiangsu Province. Journal of Plant Resources and Environment 17: 55- 60.

Li Y (1998) China weeds. Agriculture Press, Beijing.

Li Z, Xie Y (2002) Invasive alien species in China. China Forestry Publishing House, Beijing.

Li B, Wang Y, He C (2007) Primary Research on Exotic Plants in Changchun Area. Journal of Changchun Normal University (Natural Science) 26: 85-88.

Lin W, Zhou G, Cheng X, Xu R (2007) Fast economic development accelerates biological invasions in China. PLoS One 2: E1208, 1-6.

Liu J, Dong M, Miao S, Li Z, Song M, et al. (2006) Invasive alien plants in China: role of clonality and geographical origin. Biological Invasions 8: 1461-1470.

Liu J, Liang S, Liu F, Wang R, Dong M (2005) Invasive alien plant species in China: regional distribution patterns. Diversity & Distributions 11: 341-347.

Liu Q, Yu M, Zhou Y (2002) A preliminary study on the invasive plants in Beijing. Journal of Beijing Normal University (Natural Science Edition) 38: 399-404.

Liu S, Qin W (2004) Study on the current status of invasive plants in Hubei province. Journal of Central China Normal University (Natural Sciences) 38: 223-227.

Liu Y, Wang Y, Liu Y, Wei H, Zhang D (2008) Analysis of the Exotic Invasive Plants in Henan Province. Journal of Chongqing Science and Technolgy College (Nature Scienc Edtion) 10: 158-160.

Long R, Shi F, Meng X, Xu X (2008) Survey and analysis of alien invasive plants in Hebei Province. Northern Hortculture 7: 171- 173.

Ma D, Yu S, Mao R (2009) Primary Study of the areal types of Field Vascular Weeds in Sichuan Province. Journal of Sichuan NormalUniversity (Natural Science) 32: 93-97.

Ministry of Agriculture of China (1992) List of Quarantine dangerous diseases, insects, weeds of Entry Plants to the Peoples' Republic of China.

Ministry of Environmental Protection of China (2003) First batch of alien invasive species list in China.

Ministry of Environmental Protection of China (2010) Second batch of alien invasive species list in China.

Qi S, Xu W (2006) Study on types, composition and distribution characteristics of alien invasive plants in Liaoning. Journal of Liaoning Forestry Science & Technology 5: 11-15.

Qiang S, Cao X (2000) Survey and analysis of exotic weeds in China. Journal of Plant Resource and Environment 9: 34-38.

Shan J, Yang F, Zheng X (2006) Exotic Plants in Hainan Province. Subtropical Plant Science 35: 39-44.

Shi S, Tian M, Liu Y (2004) Investigation and study of the alien Invasive plants in Chongqing. Journal of Southwest China Normal University (Natural Science Edition) 29: 863-866.

Shi Y, Xie S, Wang H (2006) Study on the Exotic Invasive Plants in Shanxi Province. Journal of Tianjin Normal University (Natural Science Edition) 26: 23-27.

Song Z, Liu T, Tan D, Zhou G (2012) Influces of two invasive plants on native plant biodiversity in Xinjiang. Xinjiang Agriculture Science 49: 2120-2126.

Soyolt, Jin F, Khasbagan (2007) A Preliminary Study on the Invasive Plants in Inner Mongolia. Journal of Inner Mongolia Normal University (Natural Science Edition) 36: 480-484.

Sun C, Wang Z, Tuli G, Li J, Cheng W (2007) Harm and control strategy of alien invasive species in Jilin. Journal of Jilin Agriculture University 29: 384 -385.

Tang S, Lu S, He C, Li X, Pan Y, Pu G (2008) The alien invasive plants in Guangxi. Guihaia 28: 775-779.

Tian J (2004) Shandong invasive alien species and their control methods. Science Press, Beijing.

Tu Y (2002) Biological invasion – alien harmufull plant in Guizhou Province. Guizhou Environment Science and Technology 8: 1-5.

Wang F, Wang R, Zhuang P. Guo Q, Li Z (2009) Present status and management strategies of alien invasive plants in Guangdong Province. Chinese Journal of Ecology 28: 2088- 2093.

Wang S, Zhang N, Yu L, Zhao R, Hao P, Li J, Jiang Y, Sha H, Liu Y, Zhang Z (2012) Distribution pattern and their influcing factors of invasive alien plants in Beijing. Acta Ecologica Sinica 32: 4618-4629.

Weber E, Li B (2008) Plant invasions in China: What is to be expected in the wake of economic development? Bioscience 58: 437-444.

Weber E, Sun S, Li B (2008) Invasive alien plants in China: diversity and ecological insights. Biological Invasions 10: 1411-1429.

Wu SH, Hsieh CF, Rejmánek M (2004) Catalogue of the naturalized flora of Taiwan. Taiwania 49, 16-31.

Wu T, Li J, Dai J, Wang R (2007) Floristic characteristics and spatial distribution patterns of alien plants in Shandong Province. Chinese Journal of Ecology 26: 489- 494.

Wu X, Luo J, Chen J, LI B (2006) Spatial patterns of invasive alien plants in China and ist relationship with environmental and anthropological factors. Journal of Plant Ecology 30: 576-584.

Wu Z, Raven PH, Hong D (1994-2007) Flora of China. Science Press & Missouri Botanical Garden Beijing & St. Louis.

Xie H, Zhang X (2012) Research on invasive alien plant in Hunan province. Modern Agriculture Science and Technolgy 5: 177-181.

Xie Y, Li Z, Gregg WP, Li D (2001) Invasive species in China – an overview. Biodiversity & Conservation 10: 1317-1341.

Xu C, Lu S (2006) The invasive plants in Yunnan. Guihaia 26: 227-234.

Xu H, Qiang S (2004) Inventory of invasive alien species in China. China Environmental Science Press, Beijing.

Xu H, Qiang S, Han Z, Guo JY, Huang Z, Sun H, He S, Ding H, Wu H, Wan F (2004) The distribution and introduction pathway of alien invasive species in China. Biodiversity Science 12: 626-638.

Xu H, Wang J, Qiang S, Wang C (2004) Study of key issues under the convention on biological diversity: Alien species invasion,· biosafety,· genetic resources. Science Press, Beijing.

Yang J, Chen H (2009) A preliminary study of alien invasive plants in Fujian Province. Subtropical Plant Science 38:47-52.

Zhang M (2006) Study on the alien plants in Anhui province. Journal of Anhui Agriculture Science 34: 5306- 5308.

Zhang M, Zhang J, Wang Y (2009) A study of the protection means and the situation of biological invasion in Zhejiang Province. Journal of Inner Mongolia Agricultural University 30: 97-100.

Zheng B, Pan L (2012) Species composition of alien invasive plants in Heilongjiang Province. Biodiversity Science 20: 231-234.
