# Supplementary material for: Plant Invasions in China – Challenges and Chances
Source: PLoS One. 2013 May 14;8(5):e64173. doi: 10.1371/journal.pone.0064173 (PMC3653845; doi:10.1371/journal.pone.0064173)
Supplement: Species List S1 — List of invasive plant species in China. (DOC) [file pone.0064173.s003.doc]

**Species list S1**

**List of invasive plant species in China**

Species reported from only a single province

**Species name Family Life form**

*Aloe vera* (L.) Burm. f. Liliaceae P

*Alyssum alyssoides* (L.) L. Brassicaceae A

*Amaranthus palmeri* S. Watson Amaranthaceae A

*Amaranthus standleyanus* Parodi ex Covas Amaranthaceae A

*Avena sterilis* (Durieu) Nyman Poaceae A

*Callisia repens* L. Commelinaceae P

*Cameraria latifolia* L. Apocynaceae T

*Conium maculatum* L. Apiaceae A

*Diplotaxis muralis* (L.) DC. Brassicaceae A

*Ehrharta erecta* Lam. Poaceae P

*Elsholtzia communis* (Collett et Hemsl.) Diels Lamiaceae P

*Etlingera elatior* (Jack) R. M. Sm. Zingiberaceae P

*Euphorbia dentata* Michx. Euphorbiaceae A

*Eutrema wasabi* (Siebold) Maxim. Brassicaceae P

*Evolvulus nummularius* (L.) L. Convolvulaceae P

*Flaveria bidentis* (L.) Kuntze Asteraceae A

*Hesperis matronalis* L. Brassicaceae A

*Lilium hansonii* Leichtlin ex D. T. Moore Liliaceae P

*Martynia annua* L. Martyniaceae P

*Orobanche brassicae* (Novopokr.) Novopokr. Orobanchaceae A

*Phalaris minor* Retz. Poaceae A

*Phalaris paradoxa* L. Poaceae A

*Pyrethrum parthenifolium* Willd. Asteraceae P

*Ranunculus sardous* Crantz Ranunculaceae A

*Ranunculus trachycarpus* Fisch. et C. A. Mey. Ranunculaceae A

*Rauvolfia cubana* A. DC. Apocynaceae S

*Reseda lutea* L. Resedaceae BA

*Solanum seaforthianum* Andrews Solanaceae P

*Tradescantia spathacea* Sw. Commelinaceae P

*Vinca minor* L. Apocynaceae P

*Voacanga africana* Stapf Apocynaceae T

*Xanthium strumarium* L. Asteraceae A

Species reported from 2-19 provinces

**Species name Family Life form**

*Acacia dealbata* Link Fabaceae T

*Acacia farnesiana* (L.) Willd. Fabaceae T

*Acacia mearnsii* De Wild. Fabaceae T

*Acanthospermum australe* (Loefl.) Kuntze Asteraceae A

*Aegilops tauschii* Coss. Poaceae A

*Aeschynomene indica* L. Fabaceae A

*Ageratina adenophora* (Spreng.) R. M.King et H. Rob. Asteraceae P

*Ageratum conyzoides* L. Asteraceae A

*Ageratum houstonianum* Mill. Asteraceae A

*Agrostemma githago* L. Caryophyllaceae A

*Alliaria petiolata* (M. Bieb.) Cavara et Grande Brassicaceae A

*Alternanthera paronychioides* A. St.-Hil. Amaranthaceae P

*Alternanthera philoxeroides* (Mart.) Griseb. Amaranthaceae P

*Alternanthera pungens* Humb. et al. Amaranthaceae A

*Amaranthus albus* L. Amaranthaceae A

*Amaranthus blitoides* S. Watson Amaranthaceae A

*Amaranthus hybridus* L. Amaranthaceae A

*Amaranthus polygonoides* L. Amaranthaceae A

*Amaranthus spinosus* L. Amaranthaceae A

*Ambrosia psilostachya* DC. Asteraceae P

*Ambrosia trifida* L. Asteraceae A

*Anredera cordifolia* (Ten.) Steenis Basellaceae P

*Anthemis arvensis* L. Asteraceae A

*Argemone mexicana* L. Papaveraceae A

*Armoracia rusticana* P. Gaertn., B. Mey. et Scherb. Brassicaceae P

*Asclepias curassavica* L. Asclepiadaceae P

*Atriplex nummularia* Lindl. Chenopodiaceae P

*Axonopus compressus* (Sw.) P. Beauv. Poaceae P

*Bidens frondosa* L. Asteraceae A

*Borreria latifolia* (AuBlume) K. Schum. Rubiaceae P

*Borreria stricta* (L. f.) G. Mey. Rubiaceae A

*Brachiaria eruciformis* (Sm.) Griseb. Poaceae A

*Brachiaria mutica* (Forssk.) Stapf Poaceae P

*Bromus catharticus* Vahl Poaceae A

*Bryophyllum pinnatum* (L. f.) Oken Crassulaceae P

*Cabomba caroliniana* A. Gray Cabombaceae P

*Cassia didymobotrya* Fresen. Fabaceae S

*Cassia floribunda* Cav. Fabaceae S

*Catharanthus roseus* (L.) G. Don Apocynaceae P

*Cenchrus calyculatus* Cav. Poaceae A

*Cenchrus echinatus* L. Poaceae A

*Cenchrus spinifex* Cav. Poaceae A

*Centaurea cyanus* L. Asteraceae A

*Chamaecrista mimosoides* (L.) Greene Fabaceae BA

*Chenopodium ambrosioides* L. Chenopodiaceae BA

*Chenopodium giganteum* D. Don Chenopodiaceae A

*Chenopodium hybridum* L. Chenopodiaceae A

*Chromolaena odorata* (L.) King & H. Rob. Asteraceae P

*Chromolaena odoratum* (L.) R. King et H. Rob. Asteraceae S

*Chrysanthemum carinatum* Schousboe Asteraceae A

*Chrysanthemum coronarium* L. Asteraceae A

*Cichorium intybus* L. Asteraceae P

*Coccinia grandis* (L.) Voigt Cucurbitaceae A

*Convolvulus arvensis* L. Convolvulaceae P

*Conyza sumatrensis* (Retz.) Walker Asteraceae BA

*Cordyline fruticosa* (L.) A. Chev. Liliaceae P

*Coronopus didymus* (L.) Sm. Brassicaceae A

*Cosmos bipinnata* Cav. Asteraceae BA

*Cosmos sulphureus* Cav. Asteraceae A

*Crassocephalum crepidioides* (Benth.) S. Moore Asteraceae A

*Cucumis bisexualis* A. M. Lu et G. C. Wang ex Lu et Z. Y. Zhang Cucurbitaceae A

*Cuscuta lupuliformis* Krock. Convolvulaceae A

*Cyclospermum leptophyllum* (Pers.) Sprague ex Britton et P. Wilson Apiaceae A

*Cyperus rotundus* L. Cyperaceae P

*Datura inoxia* Mill. Solanaceae A

*Deutzia crenata* Siebold et Zucc. Saxifragaceae S

*Digitalis purpurea* L. Scrophulariaceae P

*Duranta erecta* L. Verbenaceae S

*Eichhornia crassipes* (Mart.) Solms Pontederiaceae P

*Erechtites hieraciifolius* (L.) Raf. ex DC. Asteraceae A

*Erigeron philadelphicus* L. Asteraceae BA

*Eryngium foetidum* L. Apiaceae BA

*Eupatorium catarium* Veldkamp Asteraceae A

*Euphorbia cyathophora* Murray Euphorbiaceae BA

*Euphorbia hirta* L. Euphorbiaceae A

*Euphorbia hypericifolia* L. Euphorbiaceae A

*Euphorbia maculata* L. Euphorbiaceae A

*Euphorbia marginata* Pursh Euphorbiaceae A

*Euphorbia prostrata* Aiton Euphorbiaceae A

*Euphorbia serpens* Kunth Euphorbiaceae A

*Gaura parviflora* Douglas Onagraceae A

*Geranium carolinianum* L. Geraniaceae A

*Gomphrena celosioides* C. Mart. Amaranthaceae A

*Helenium autumnale* L. Asteraceae P

*Heliotropium europaeum* L. Boraginaceae A

*Herissantia crispa* (L.) Brizicky Malvaceae BA

*Hordeum jubatum* L. Poaceae A

*Hylocereus undatus* (Haw.) Britton et Rose Cactaceae P

*Hyptis brevipes* Poit. Lamiaceae A

*Hyptis rhomboidea* Mart. et Galeotti Lamiaceae A

*Hyptis suaveolens* (L.) Poit. Lamiaceae A

*Ipomoea alba* L. Convolvulaceae P

*Ipomoea cairica* (L.) Sweet var. gracillima (Collett et Hemsl.) C. Y. Wu et Li Convolvulaceae P

*Ipomoea carnea* Jacquem. subsp. fistulosa (Mart. ex Choisy) D. F. Austin Convolvulaceae S

*Ipomoea indica* (Burm.) Merr. Convolvulaceae A

*Jatropha curcas* L. Euphorbiaceae P

*Lactuca seriola* Torner Asteraceae A

*Lantana camara* L. Verbenaceae S

*Lepidium campestre* (L.) R. Br. Brassicaceae A

*Lepidium densiflorum* Schrad. Brassicaceae A

*Lepidium perfoliatum* L. Brassicaceae BA

*Lepidium sativum* L. Brassicaceae A

*Leucaena leucocephala* (Lam.) de Wit Fabaceae T

*Leucanthemum vulgare* Tourn. ex Lam. Asteraceae P

*Lobularia maritima* (L.) Desv. Brassicaceae P

*Lolium multiflorum* Lamk. Poaceae A

*Lolium persicum* Boiss. et Hoh. Poaceae A

*Lotus corniculatus* L. Fabaceae P

*Macfadyena unguis-cati* (L.) A.H. Gentry Bignoniaceae P

*Malvastrum coromandelianum* (L.) Gürcke Malvaceae P

*Medicago polymorpha* L. Fabaceae BA

*Melinis repens* (Willd.) Zizka Poaceae P

*Mikania micrantha* Kunth Asteraceae P

*Mimosa bimucronata* (DC.) Kuntze Fabaceae P

*Mimosa invisa* Mart. ex Colla Fabaceae P

*Mimosa pudica* L. Fabaceae P

*Mirabilis jalapa* L. Nyctaginaceae A

*Narcissus tazetta* L. Amaryllidaceae P

*Nerium oleander* L. Apocynaceae T

*Nicandra physalodes* (L.) Gaertn. Solanaceae A

*Nymphaea alba* L. Nymphaeaceae P

*Ochrosia elliptica* Labill. Apocynaceae T

*Oenothera biennis* L. Onagraceae P

*Oenothera rosea* L'Hér. ex Aitch. Onagraceae P

*Opuntia ficus-indica* (L.) Mill. Cactaceae S

*Opuntia monacantha* (Willd.) Haw. Cactaceae S

*Opuntia stricta* (Haw.) Haw. Cactaceae S

*Orobanche coerulescens* Stephan Orobanchaceae P

*Panicum maximum* Jacquin Poaceae P

*Panicum repens* L. Poaceae P

*Papaver nudicaule* L. Papaveraceae P

*Parthenium hysterophorus* L. Asteraceae A

*Parthenocissus quinquefolia* (L.) Planch. Vitaceae A

*Paspalum conjugatum* Bergius Poaceae P

*Paspalum* *dilatatum* Poir. Poaceae P

*Paspalum fimbriatum* Kunth Poaceae A

*Passiflora caerulea* L. Passifloraceae P

*Passiflora foetida* L. Passifloraceae P

*Pennisetum glaucum* (L.) R. Br. Poaceae A

*Pennisetum polystachion* (L.) Schultes Poaceae P

*Pennisetum purpureum* Schumach. Poaceae P

*Peperomia pellucida* (L.) Kunth Piperaceae A

*Phleum pratense* L. Poaceae P

*Physalis peruviana* L. Solanaceae P

*Physalis philadelphica* Lam. Solanaceae A

*Phytolacca americana* L. Phytolaccaceae P

*Pilea microphylla* (L.) Liebm. Urticaceae A

*Pistia stratiotes* L. Araceae A

*Pittosporum tobira* (Thunb.) W. T. Aiton Pittosporaceae T

*Plantago aristata* Michx. Plantaginaceae A

*Plantago lanceolata* L. Plantaginaceae P

*Plantago virginica* L. Plantaginaceae A

*Poa compressa* L. Poaceae P

*Pseudelephantopus spicatus* (Juss. ex Aubl.) Gleason Asteraceae A

*Psidium guajava* L. Myrtaceae T

*Ranunculus arvensis* L. Ranunculaceae A

*Ranunculus muricatus* L. Ranunculaceae A

*Raphanus raphanistrum* L. Brassicaceae A

*Ribes multiflorum* Kit. ex Roem. et Schult. Saxifragaceae S

*Ribes nigrum* L. Saxifragaceae S

*Salix fragilis* L. Salicaceae T

*Scoparia dulcis* L. Scrophulariaceae A

*Senecio dubitabilis* C. Jeffrey et Y. L. Chen Asteraceae A

*Senecio vulgaris* L. Asteraceae A

*Senna alata* (L.) Roxb. Fabaceae S

*Senna hirsuta* (L.) H. S. Irwin et Barneby Fabaceae S

*Senna occidentalis* (L.) Link Fabaceae S

*Senna sophera* (L.) Roxb. Fabaceae S

*Setaria palmifolia* (J. Konig) Stapf Poaceae P

*Setaria parviflora* (Poir.) Kerguélen Poaceae P

*Sicyos angulatus* L. Cucurbitaceae A

*Sida rhombifolia* L. Malvaceae P

*Silene vulgaris* (Moench) Garcke Caryophyllaceae P

*Sinapis alba* L. Brassicaceae A

*Sisymbrium altissimum* L. Brassicaceae A

*Sisymbrium orientale* L. Brassicaceae A

*Smallanthus uvedalius* (L.) Mack. ex Small Asteraceae P

*Solanum aculeatissimum* Jacquem. Solanaceae A

*Solanum capsicoides* All. Solanaceae P

*Solanum chrysotrichum* Schltdl. Solanaceae S

*Solanum erianthum* D. Don Solanaceae S

*Solanum rostratum* Dunal Solanaceae A

*Solanum sisymbriifolium* Lam. Solanaceae A

*Solanum torvum* Sw. Solanaceae P

*Solidago altissima* L. Asteraceae P

*Solidago canadensis* L. Asteraceae P

*Soliva anthemifolia* (Juss.) R. Br. Asteraceae A

*Sonchus arvensis* L. Asteraceae P

*Sonneratia apetala* Buch.-Ham. Lythraceae T

*Sorghum halepense* (L.) Persoon Poaceae P

*Sorghum sudanense* (Piper) Stapf Poaceae A

*Spartina alterniflora* Loisel. Poaceae P

*Spartina anglica* C. E. Hubb. Poaceae P

*Spergula arvensis* L. Caryophyllaceae A

*Sphagneticola trilobata* (L.) Pruski Asteraceae P

*Stachytarpheta jamaicensis* (L.) Vahl Verbenaceae P

*Stellaria pallida* (Dumort.) Crép. Caryophyllaceae P

*Striga asiatica* (L.) Kuntze Scrophulariaceae A

*Symphyotrichum subulatum* (Michx.) G.L. Nesom Asteraceae A

*Synedrella nodiflora* (L.) Gaertn. Asteraceae A

*Tagetes erecta* L. Asteraceae A

*Tagetes patula* L. Asteraceae A

*Talinum paniculatum* (Jacq.) Gaertn. Portulacaceae P

*Tithonia diversifolia* A. Gray Asteraceae BA

*Tradescantia zebrina* Bosse Commelinaceae P

*Tridax procumbens* L. Asteraceae P

*Trifolium hybridum* L. Fabaceae P

*Urochloa decumbens* (Stapf) R. Webster Poaceae A

*Veronica arvensis* L. Scrophulariaceae A

*Veronica hederifolia* L. Scrophulariaceae A

*Veronica peregrina* L. Scrophulariaceae A

*Vetiveria zizanioides* (L.) Nash Poaceae P

*Waltheria indica* L. Sterculiaceae S

*Xanthium spinosum* L. Asteraceae A

*Zinnia peruviana* (L.) L. Asteraceae P

Species reported from 20 or more provinces

**Species name Family Life form**

*Abutilon theophrasti* Medik. Malvaceae A

*Agave americana* L. Amaryllidaceae P

*Amaranthus blitum* L. Amaranthaceae A

*Amaranthus caudatus* L. Amaranthaceae A

*Amaranthus cruentus* L. Amaranthaceae A

*Amaranthus retroflexus* L. Amaranthaceae A

*Amaranthus tricolor* L. Amaranthaceae A

*Amaranthus viridis* L. Amaranthaceae A

*Ambrosia artemisiifolia* L. Asteraceae A

*Avena fatua* L. Poaceae A

*Bidens pilosa* L. Asteraceae A

*Brassica juncea* (L.) Czern. Brassicaceae A

*Brassica rapa* L. Brassicaceae A

*Buchloe dactyloides* (Nutt.) Engelm. Poaceae P

*Cannabis sativa* L. Cannabaceae A

*Capsella bursa-pastoris* (L.) Medik. Brassicaceae A

*Capsicum annuum* L. Solanaceae BA

*Conyza bonariensis* (L.) Cronq. Asteraceae A

*Conyza canadensis* (L.) Cronq. Asteraceae A

*Coreopsis grandiflora* Hogg ex Sweet Asteraceae P

*Coreopsis lanceolata* L. Asteraceae P

*Coreopsis tinctoria* Nutt. Asteraceae BA

*Coriandrum sativum* L. Apiaceae A

*Cucumis melo* L. Cucurbitaceae A

*Cuscuta europaea* L. Convolvulaceae A

*Cuscuta japonica* Choisy Convolvulaceae A

*Datura metel* L. Solanaceae A

*Datura stramonium* L. Solanaceae A

*Daucus carota* L. Apiaceae A

*Digitaria ciliaris* (Retz.) Koeler Poaceae A

*Echinochloa crus-galli* (L.) P. Beauv. Poaceae A

*Eleusine indica* (L.) Gaertn. Poaceae A

*Erigeron annuus* (L.) Pers. Asteraceae A

*Euphorbia helioscopia* L. Euphorbiaceae A

*Euphorbia lathyris* L. Euphorbiaceae A

*Galinsoga parviflora* Cav. Asteraceae A

*Helianthus annuus* L. Asteraceae A

*Helianthus tuberosus* L. Asteraceae P

*Hibiscus trionum* L. Malvaceae A

*Humulus scandens* (Lour.) Merr. Cannabaceae BA

*Ipomoea nil* (L.) Roth Convolvulaceae A

*Lemna aequinoctialis* Welw. Lemnaceae P

*Lepidium virginicum* L. Brassicaceae A

*Lolium perenne* L. Poaceae P

*Lolium temulentum* L. Poaceae A

*Medicago sativa* L. Fabaceae P

*Melilotus albus* Medik. Fabaceae A

*Melilotus officinalis* (L.) Pall. Fabaceae A

*Nasturtium officinale* R. Br. Brassicaceae P

*Oxalis corymbosa* DC. Oxalidaceae P

*Pharbitis purpurea*(L.)Voigt Convolvulaceae A

*Prunella vulgaris* L. Lamiaceae P

*Rhus typhina* L. Anacardiaceae T

*Ricinus communis* L. Euphorbiaceae A

*Robinia pseudoacacia* L. Fabaceae T

*Rosmarinus officinalis* L. Lamiaceae S

*Salvia coccinea* Buc'hoz ex Etl. Lamiaceae A

*Senna tora* (L.) Roxb. Fabaceae A

*Silybum marianum* (L.) Gaertn. Asteraceae A

*Sinapis arvensis* L. Brassicaceae A

*Sonchus asper* (L.) Hill Asteraceae A

*Sonchus oleraceus* L. Asteraceae A

*Symphytum officinale* L. Boraginaceae P

*Trifolium incarnatum* L. Fabaceae A

*Trifolium pratense* L. Fabaceae P

*Trifolium repens* L. Fabaceae P

*Ulex europaeus* L. Fabaceae S

*Vaccaria hispanica* (Mill.) Rauschert Caryophyllaceae A

*Veronica persica* Poir. Scrophulariaceae A

*Veronica polita* Fries Scrophulariaceae BA

*Zephyranthes candida* (Lindl.) Herb. Amaryllidaceae P

*Zephyranthes carinata* Herb. Amaryllidaceae P

Life forms: A: annual herb; BA: biannual herb; P: perennial herb; S: shrub; T: tree
